# Clinical Characteristics, MRI Findings, Disease Progression, and Management of Neuro-Behçet’s Disease: A Retrospective Study in Lebanon

**DOI:** 10.3390/jcm14082543

**Published:** 2025-04-08

**Authors:** Nadia Chamoun, Martine Elbejjani, Nabil K. El Ayoubi, Taha Hatab, Dana Hazimeh, Michael Ibrahim, Mira Merashli

**Affiliations:** 1 Department of Internal Medicine, American University of Beirut Medical Center, Beirut 1107 2020, Lebanon; nadia.chamoun@hotmail.com (N.C.); me158@aub.edu.lb (M.E.); tmh13@mail.aub.edu (T.H.); dhazime1@jhmi.edu (D.H.); ibrahemm@upmc.edu (M.I.); 2 Clinical Research Institute, Faculty of Medicine, American University of Beirut Medical Center, Beirut 1107 2020, Lebanon; 3 Department of Neurology, American University of Beirut Medical Center, Beirut 1107 2020, Lebanon; nke08@mail.aub.edu; 4 Division of Rheumatology, Department of Internal Medicine, American University of Beirut Medical Center, Beirut 1107 2020, Lebanon

**Keywords:** Behçet’s Disease, Neuro-Behçet, neurologic involvement, Middle East, magnetic resonance imaging

## Abstract

**Background**: Behçet’s Disease (BD) is a complex vasculitis affecting multiple organ systems, with Neuro-Behçet’s Disease (NBD) representing a rare yet severe manifestation. Data on NBD are limited, particularly in Middle Eastern populations. **Methods**: This retrospective observational study, spanning from 2000 to 2021, involved 262 BD patients at a tertiary medical center in Lebanon. NBD was diagnosed based on International Consensus Recommendation diagnostic criteria. Clinical data, including demographics, manifestations, inflammatory blood markers, genetics, and treatments, were collected. The modified Rankin Scale (mRS) was used to assess disease severity. **Results**: Among the cohort, 27 (10.3%) had NBD, with headaches, weakness, and dizziness as the most common presenting symptoms. The prevalence of NBD was similar across genders, which differs from some regional studies. HLA-B51 positivity was found in 50 out of 60 (83.3%) tested BD patients. Parenchymal NBD cases exhibited greater disease severity than non-parenchymal cases, with female patients experiencing a more severe course compared to males. Elevated inflammatory markers (CRP and ESR) were more common in patients with severe NBD. Corticosteroids and colchicine were the most commonly used therapies overall, while patients with better disease severity were more frequently prescribed methotrexate, mycophenolate, cyclophosphamide, adalimumab, and rituximab. An analysis of disease progression showed that at presentation, 57.1% (*n* = 12) of NBD patients had mild to moderate disability, which increased to 76.2% (*n* = 16) at the last follow-up, including 10 patients who showed an improvement in their mRS score. **Conclusions**: This study provides valuable insights into the prevalence and clinical characteristics of NBD in a Middle Eastern population. These findings enhance our understanding of NBD in the Middle East, highlighting the need for further research to improve diagnosis and management.

## 1. Introduction

Behçet’s Disease (BD) is a chronic, multisystem vasculitis affecting small-, medium-, and large-sized blood vessels [1]. It is most commonly seen in regions along the historic “Silk Route”, from the Mediterranean to the Far East, with particularly high prevalence in Turkey and Iraq compared to Western nations [2]. BD typically presents in the third or fourth decades of life and is characterized by a wide range of clinical features, including oral and genital ulcers, uveitis, arthritis, vascular involvement, and various skin lesions. Its diagnosis remains clinical, as no specific biomarkers or pathognomonic imaging findings exist. [2,3]. Among the existing diagnostic tools, the International Criteria for Behçet’s Disease (ICBD) is the most sensitive [4] while the International Study Group (ISG) criteria are the most specific and commonly used [5].

One of the most severe and potentially disabling manifestations of BD is neurological involvement, referred to as Neuro-Behçet’s Disease (NBD), with a reported prevalence ranging from 2.2% to 50% [6]. NBD usually develops within the first five years of BD onset, and it is more commonly observed in male patients in the Middle East [7]. It primarily affects the central nervous system (CNS) and is classified into parenchymal and non-parenchymal forms, with the former being more common. [1] Diagnosing NBD is particularly challenging due to its rarity, non-specific symptoms, and overlap with other neurological disorders. Diagnosis typically requires a combination of neuroimaging, cerebrospinal fluid (CSF) analysis, clinical evaluation, and, in some cases, nervous tissue biopsy [1].

Despite its clinical significance, evidence-based guidance on NBD remains limited, particularly regarding the distinction between parenchymal and non-parenchymal subtypes. This study aims to address this gap by reporting the clinical characteristics, subtype distribution, and inflammatory profiles of patients with NBD at a tertiary care center in Lebanon. By doing so, we hope to contribute to the growing but still limited body of literature on the spectrum and severity of NBD in the Middle Eastern population.

## 2. Materials and Methods

This single-center, retrospective observational study was conducted on patients diagnosed with BD at the American University of Beirut Medical Center (AUBMC), a tertiary care facility in Beirut, Lebanon. The study covered the period from January 2000 to January 2021, utilizing data collected retrospectively from medical records. The institutional review board at the AUBMC approved the study (Approval Code: BIO-2020-0262; Approval Date: 20 September 2021) and granted a waiver of informed consent.

Adults aged 18 years and older diagnosed with BD at the AUBMC, according to the 1990 ISG BD criteria [5] and/or the new ICBD [4], were included in the study. NBD diagnosis was made by specialized rheumatologists and neurologists at the AUBMC following the International Consensus Recommendation (ICR) [1]. Patients with neurological symptoms attributed to confirmed diagnoses other than NBD were excluded. Participants were stratified into two groups: BD without neurological involvement (BD-NI) and those diagnosed with NBD.

Further stratification among NBD patients was performed based on disease severity using the modified Rankin Scale (mRS) score at the last follow-up visit. The mRS is a validated tool used to assess the degree of disability in daily activities, as detailed in Table S1 [8]. The mRS scores categorized NBD patients into mild-to-moderate (scores 0–3) or severe (scores 4–6) groups.

Clinical data were retrieved from patient records, including demographics, manifestations, NBD types, diagnosis dates, disease duration, time to NBD onset, inflammatory blood markers, HLA-B51 status, and MRI findings.

NBD types were classified as parenchymal and non-parenchymal based on MRI findings, which were analyzed and interpreted by highly trained neuroradiologists at AUBMC. BD duration was defined as the time from diagnosis to the last medical examination, while time to NBD onset referred to the period from BD diagnosis to the onset of neurologic symptoms. Patients were followed up from the date of their BD diagnosis till their last follow up at the AUBMC. Study data were collected and managed using the Research Electronic Data Capture (REDCap) tool, hosted at the AUBMC [9,10].

Data management and statistical analysis were performed using SPSS Version 27. Descriptive analysis was applied to the clinical and radiological variables specified in the study. Categorical variables were reported as frequencies and percentages, while continuous variables were presented as means ± standard deviations. Due to the limited sample size of NBD patients, we chose to use descriptive statistics and did not conduct hypothesis testing.

## 3. Results

A total of 262 patients diagnosed with BD at the AUBMC from January 2000 until January 2021 were included in the study. The mean age of all cases was 44.0 ± 15.3 years, with 126 or 48.1% being female, and 27 patients (10.3%) were diagnosed with NBD.

### 3.1. Behçet’s Disease Without Neurological Involvement vs. Neuro-Behçet’s Disease

Table 1 summarizes the baseline characteristics, clinical features, and genetic and inflammatory markers of the BD-NI and NBD groups. The mean age of NBD patients was 47.5 ± 13.5 years, with 14 (51.9%) being female.

Although no formal hypothesis testing was performed, notable trends emerged. Compared to the BD-NI group, NBD patients had higher rates of genital ulcers (19 patients or 70.4% vs. 125 patients or 53.2%), arthritis or arthralgia (14 patients or 51.9% vs. 102 patients or 43.4%), and cutaneous involvement (11 patients or 40.7% vs. 59 patients or 25.1%). Neurological symptoms were markedly more frequent in the NBD group, including headaches (24 patients or 88.9% vs. 34 patients or 14.5%), weakness (17 patients or 63% vs. 12 patients or 5.1%), dizziness (11 patients or 40.7% vs. 9 patients or 3.8%), unsteadiness (9 patients or 33.3% vs. 5 patients or 2.1%), cognitive/behavioral changes (7 patients or 25.9% vs. 1 patient or 0.4%), paresthesia (6 patients or 22.2% vs. 8 patients or 3.4%), seizure/epilepsy (5 patients or 18.5% vs. 3 patients or 1.3%), and diplopia (3 patients or 11.1% vs. 3 patients or 1.3%). Patients in the BD-NI group who presented with neurological symptoms did not meet diagnostic criteria for NBD.

HLA-B51 genetic testing was available for 60 patients (22.9%), of whom 50 (83.3%) tested positive. Among those, 46 out of 55 (83.6%) belonged to the BD-NI group and 4 out of 5 (80.0%) to the NBD group. Inflammatory markers were measured before and after treatment, with CRP > 2.5 mg/L and ESR > 20 mm/hr considered elevated. Before treatment, elevated CRP was observed in 25 out of 42 patients (59.5%): 17 of 29 in the BD-NI group and 8 of 13 in the NBD group. Elevated ESR was noted in 22 out of 40 patients (55.0%): 14 of 25 in BD-NI and 8 of 15 in NBD. Of the 22 patients with both pre- and post-treatment CRP data, the levels remained normal in 12 (54.5%) (9 BD-NI and 3 NBD), normalized in 5 (22.7%) (3 BD-NI and 2 NBD), and remained elevated in 5 (22.7%) (2 BD-NI and 3 NBD). For ESR, 17 patients had both pre- and post-treatment values. The levels remained normal in seven (five BD-NI and two NBD), normalized in six (two BD-NI and four NBD), and stayed elevated in four (one BD-NI and three NBD).

Out of 262 patients, 246 (93.9%) received treatment for Behçet’s Disease. Table 2 summarizes the treatments received among patients with NBD compared to those without neurological involvement. *Colchicine* was the most commonly used medication overall (71.4%), particularly in the BD-NI group (72.8%), while *corticosteroids* were the most frequently prescribed treatment in the NBD group (77.8%). Immunosuppressants were more commonly used among NBD patients, including azathioprine (48.1% vs. 17%), methotrexate (22.2% vs. 7.2%), mycophenolate mofetil (18.5% vs. 1.3%), and cyclophosphamide (18.5% vs. 0.4%). Infliximab (11.8%) and adalimumab (14.1%) were the most commonly used biologics, more frequently in NBD.

Cutaneous involvement among NBD patients consisted of five cases of pseudo-folliculitis, five cases of erythema nodosum, and one case of erythematous skin rash. Additionally, uveitis manifestations in the NBD group included two cases of anterior uveitis, one case of posterior uveitis, and five cases of panuveitis, as outlined in Table 3.

### 3.2. NBD Stratification by mRS Score

Table 4 outlines the clinical features stratified by the mRS severity score. Most NBD patients (20 patients or 74.1%) had mild to moderate disability (scores 0–3), while 7 (25.9%) had severe disability (scores 4–6). The mean age of BD onset was 32.3 ± 13.3 years, and the time to NBD was 4.6 ± 7.7 years. In the mild-to-moderate group, recurrent oral (17 patients or 85%) and genital (15 patients or 75.0%) ulcers were the most common non-neurological manifestations, followed by joint (10 patients or 50.0%) and cutaneous (8 patients or 40.0%) involvement. Uveitis occurred in seven (35.0%) patients, while gastrointestinal involvement was observed in six (30.0%). In the severe group, oral ulcers (six patients or 85.7%) were also predominant, followed by uveitis (five patients or 71.4%) and genital ulcers, joint involvement, and cutaneous manifestations (four patients or 57.1% each). Deep-vein thrombosis exhibited the lowest occurrence among manifestations in both the mild-to-moderate (two patients or 10%) and severely disabled groups (one patient or 14.3%).

Regarding neurological manifestations, headaches (19 patients or 95.0% vs. 5 patients or 71.4%) and weakness (13 patients or 65.0% vs. 4 patients or 57.1%) were highly prevalent in both NBD severity. Patients with severe NBD exhibited additional symptoms such as dizziness, unsteadiness, and cognitive/behavioral changes (three patients or 42.9% each), followed by diplopia, paresthesia, and seizures (two patients or 28.6% each), with no occurrences of dysphagia or psychosis. In the mild-to-moderate group, dizziness occurred at a rate similar to the severe group (eight patients or 40.0%), followed by unsteadiness (six patients or 30.0%), paresthesia (four patients or 20.0%), cognitive/behavioral changes (four patients or 20.0%), seizures (three patients or 15.0%), diplopia (two patients or 10.0%), psychosis (two patients or 10.0%), and only one instance of dysphagia (5.0%).

Pre-treatment CRP levels were available for 13 patients: elevated CRP was observed in 4 out of 7 patients (57.1%) in the mild-to-moderate group and in a higher proportion, 4 out of 6 patients (66.7%), in the severe group. Similarly, among the 15 patients with available pre-treatment ESR values, elevated levels were found in 4 out of 9 (44.4%) in the mild-to-moderate group and in a higher 4 out of 6 (66.7%) in the severe group. Among those with both pre- and post-treatment CRP data, the levels remained elevated in 1 out of 4 (25.0%) in the mild-to-moderate group and a higher 2 out of 4 (50.0%) in the severe group. CRP normalized in one patient in each group (25.0%) and remained normal in 2 out of 4 (50.0%) in the mild-to-moderate group and 1 out of 4 (25.0%) in the severe group. As for ESR, levels remained elevated in 1 out of 5 patients (20.0%) in the mild-to-moderate group versus a higher 2 out of 4 (50.0%) in the severe group. ESR normalized in 2 out of 5 (40.0%) and 2 out of 4 patients (50.0%) in the mild-to-moderate and severe groups, respectively. It remained normal in 2 out of 5 patients (40.0%) in the mild-to-moderate group.

Compared to the severe disease group, the mild-to-moderate disease group was more frequently prescribed the following medications: methotrexate (25.0% vs. 14.3%), mycophenolate (20.0% vs. 14.3%), cyclophosphamide (20.0% vs. 14.3%), rituximab (10.0% vs. 0.0%), adalimumab (15.0% vs. 0.0%), and tocilizumab (5.0% vs. 0.0%). On the other hand, medications more commonly prescribed in the severe disease group included steroids (85.7% vs. 75.0%), colchicine (71.4% vs. 55.0%), infliximab (28.6% vs. 15.0%), azathioprine (57.7% vs. 45.0%), and cyclosporine (14.3% vs. 5.0%).

### 3.3. Parenchymal Versus Non-Parenchymal NBD Analysis

Table 5 provides a comprehensive overview of the baseline characteristics, neurological and non-neurological symptoms, and severity of neurological involvement in patients with parenchymal and non-parenchymal NBD. Among our NBD cohort (*n* = 27), 8 cases (33.3%) were classified as parenchymal disease, while 16 cases (66.7%) were categorized as non-parenchymal. Three NBD patients had missing MRI results and were consequently unclassified.

Both parenchymal and non-parenchymal NBD groups exhibited recurrent oral ulcers (7 patients or 87.5% vs. 13 patients or 81.3%) and genital ulcers (5 patients or 62.5% vs. 12 patients or 75.0%) as the most prevalent non-neurological findings. In parenchymal NBD, there was a notable prevalence of uveitis (five patients or 62.5%) and cutaneous involvement (four patients or 50.0%). Non-parenchymal NBD cases presented with high rates of joint involvement (10 patients or 62.5%) and cutaneous manifestations (7 patients or 43.8%), followed by uveitis (6 patients or 37.5%), gastrointestinal symptoms (5 patients or 31.3%), and only 2 cases of deep vein-thrombosis (DVT).

### 3.4. MRI Findings

MRI findings stratified by severity and subtype are detailed in Table 4 and Table 6, respectively. Among patients with mild to moderate disability, brainstem and periventricular lesions were most common (six patients or 30.0%, each), while severe cases showed higher involvement of the basal ganglia (four patients or 57.1%) and thalamus (three patients or 42.9%). In both groups, ischemic infarcts were frequently observed (seven patients or 35.0% in mild to moderate and two patients or 28.6% in severe cases). MRI findings in non-parenchymal NBD revealed ischemia or infarcts as the most common (nine patients or 56.3%), followed by sinus or vein thrombosis (five patients or 31.3%), and meningeal enhancement (four patients or 25.0%). Less frequent findings included aneurysms and cerebral bleeding (two patients or 12.5%, each). In parenchymal NBD, brainstem lesions were most common (six patients or 75.0%), followed by the thalamus (five patients or 62.5%), basal ganglia (three patients or 37.5%), and internal capsule (three patients or 37.5%). Other affected areas included the periventricular region (two patients or 25.0%), spinal cord (one case), and hypothalamus (one case).

### 3.5. Disease Progression in NBD

**Figure 1:**
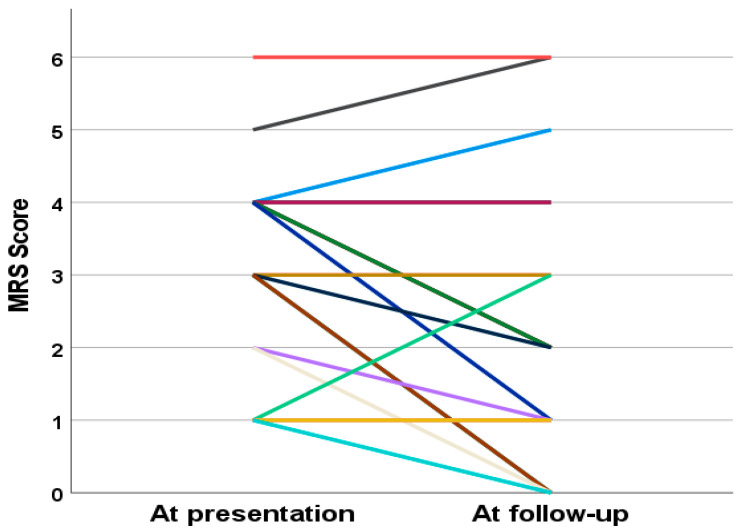
Line plot representing individual changes in modified Rankin Scale (mRS) scores from presentation to follow-up for each subject. Each color corresponds to a different subject (*n* = 21). Each color= each line represents a patient.

The mRS scores were recorded at two time points—at presentation and at the last follow-up—in 21 NBD patients. At presentation, a majority of patients had mild to moderate severity (57.1%, *n* = 12), while 42.9% (*n* = 9) had severe disease. At the last follow-up, 76.2% of patients (*n* = 16) had mild to moderate disease, and 23.8% (*n* = 5) had severe disease. A more detailed breakdown of the mRS scores at both time points is provided in Table 7. The patient-by-patient analysis in Figure 1 reveals that 8 patients (38.1%) had no change in their mRS score, 10 patients (47.6%) experienced an improvement in severity, and 3 patients (14.3%) had a worsening of their score.

## 4. Discussion

In this 20-year study, we analyzed the clinical and radiological characteristics of 262 BD patients, with 27 cases diagnosed as NBD. The relatively low number of NBD cases raises concerns about potential underdiagnosis, which might be due to the misattribution of symptoms to other neurological conditions [11]. Additionally, Lebanon’s recent economic crisis, as reflected in its reclassification as a lower–middle-income nation by the World Bank [12], could have impacted patient follow-up and the affordability of diagnostic tests such as MRI, CSF analysis, and biopsies.

The Middle East reports a higher prevalence of NBD compared to other regions, which may be influenced by variations in BD incidence, population characteristics, and diagnostic criteria [13]. Three prospective studies from Turkey [14], Iran [15], and Iraq [6] found neurological involvement in 5.3% to 14.3% of BD patients, with a pooled average of 9.4% (43 out of 459) [16]. Our institution reported a similar prevalence of 10.3%.

Our study found that the average age of onset for NBD was 38.0 ± 13.6 years, consistent with the reported range of 20–40 years [7,16]. NBD typically manifests 3–6 years after systemic BD symptoms [7], with 6% experiencing neurological involvement as the initial BD symptom [17]. We observed a time to NBD of 4.6 ± 7.7 years, aligning with the existing literature.

Studies from Turkey (155 men/45 women) [18], Saudi Arabia (42/10) [19], Tunisia (61/14 [20] and 78/43) [21], and the UK (31/19) [22] have generally reported a male predominance in NBD cases. In contrast, a study from southwest England and south Wales (no gender difference) [23], as well as a Korean study (9/12) [24], did not show this gender trend. Our cohort had a balanced gender ratio (13 men/14 women). Previous studies on gender impact in NBD prognosis have yielded mixed results, with some suggesting increased morbidity and mortality in males [18,21,25], and others finding no significant gender-based differences [7,26,27]. In our study, 74.1% of NBD cases were classified as mild to moderate and 25.9% as severe. Interestingly, we found that female patients experienced a more severe disease course than their male counterparts, with 35.7% of female NBD patients affected by severe disease compared to just 15.2% of males.

Oral ulcers are a common systemic symptom in BD, with genital ulcers also frequently observed. The prevalence of genital ulcers varies widely, generally estimated between 60 and 80% [16,28]. Our study found an 81.7% rate for oral ulcers and 55% for genital ulcers. Notably, our reported prevalence of genital ulcers is lower than that observed in many developed countries, such as France (73%) and Sweden (76%), but is similar to findings from Iran (64.6%) [29]. This discrepancy may reflect our small sample size, under-reporting due to stigma, or the lack of a dedicated Behçet’s clinic at AUBMC for systematic assessment.

Uveitis is a common ocular manifestation in BD, with prevalence rates ranging from approximately 50% in multidisciplinary settings to over 90% in specialized ophthalmology departments [30]. Our study documented a uveitis rate of 42.7%, which is comparable to the prevalence reported in multidisciplinary settings but lower than the rates observed in specialized ophthalmology departments.

Cutaneous manifestations in BD vary widely, ranging from 48 to 88% [31]. Our study reported a lower prevalence of 26.7%, with a higher occurrence of 40.7% among NBD patients compared to 25.1% in non-neurological BD patients. Among NBD patients with cutaneous involvement, pseudo-folliculitis and erythema nodosum were the most common types.

Neurological symptoms, such as headache, diplopia, dizziness, unsteadiness, seizures, weakness, paresthesia, and cognitive or behavioral changes, were more prevalent in NBD patients compared to those without NBD. Headaches were the most common neurological symptom in NBD patients, with a prevalence of 88.9%, which aligns with the previously reported prevalence of 70% [16].

Previous studies have reported an HLA-B51 prevalence of 50–80% in BD patients [32], with higher rates in Turkey (84%) [33] compared to European countries (Germany: 36.2%, Italy: 57.4%, and Spain: 36.2%) [32]. In our study, 83.3% of BD patients with an available HLA status were HLA-B51 positive, aligning with the higher prevalence of this gene along the Silk Route.

Pre-treatment levels of CRP and ESR were elevated in a substantial proportion of patients in both the NBD and BD-NI groups, with no substantial difference between the two groups. However, an elevated CRP and ESR were more frequently observed in the severe NBD group compared to the mild-to-moderate group, suggesting that higher levels of these inflammatory markers correlate with greater disease severity. This finding aligns with the existing literature, where elevated inflammatory markers have been shown to reflect active disease and may be indicative of disease severity in various autoimmune and vasculitic conditions, including BD and NBD specifically [34,35]. Interestingly, when examining post-treatment changes, both CRP and ESR showed significant normalization. Comparing NBD vs. BD-NI, the CRP and ESR decreased in 62.5% of NBD patients and 58.0% of BD-NI patients, which is relatively similar, suggesting that treatment effectively reduced inflammation in both groups [35]. However, when comparing the mild-to-moderate vs. severe disease groups, a greater reduction in both CRP and ESR was observed in the mild-to-moderate group, with a 75.0% decrease in CRP and ESR compared to only a 50.0% decrease in the severe group. This indicates that patients with less severe disease had a more robust response to treatment, with a greater reduction in inflammatory markers.

NBD can be categorized into parenchymal and non-parenchymal types, with varying prevalences across populations. Studies from Turkey and Japan reported parenchymal NBD in 60.6% and 57% of cases, respectively, and non-parenchymal NBD in 39.4% and 43% [14,18,36]. In our study, 66.7% of NBD cases were non-parenchymal, and 33.3% were parenchymal. Multiple studies suggested a better prognosis for non-parenchymal CNS involvement compared to parenchymal involvement [18]. Our study supports this, with a higher proportion of severe disease observed in parenchymal NBD (37.5% vs. 18.75%) compared to non-parenchymal NBD.

Regarding MRI findings in NBD, cerebral venous thrombosis (CVT) is reported in 0% to 9.1% of cases in Far Eastern and European countries but up to 42% in the Middle East [16]. Our study found a similar prevalence of 31.3%. Notably, the presence of aneurysms and cerebral bleeding (12.5%) in this sample is also noteworthy, as these complications are relatively rare but can be severe and life threatening [37,38]. Previous studies have identified brainstem lesions as the most common type of parenchymal NBD, with a prevalence of 67.8% [39], consistent with our finding of 75.0%. This study also reported 41.4% for basal ganglia lesions and 26.4% for thalamic lesions, mirroring our population with 37.5% basal ganglia involvement and a higher rate of 62.5% thalamic lesions. Spinal cord involvement was observed in 12.5% of our cases, aligning with the 10% prevalence reported elsewhere [19,22,23].

Corticosteroids and colchicine were the most commonly used medications in both the BD-NI and NBD groups. Immunosuppressants, including azathioprine, methotrexate, mycophenolate mofetil, and cyclophosphamide, and biologic therapies, such as infliximab, were used more frequently in NBD, reflecting a tailored treatment approach aimed at managing more severe or systemic disease manifestations [40].

The mRS scores recorded at presentation and at the last follow-up revealed a favorable trend in disease progression. At presentation, the majority of patients had mild to moderate severity (57.1%), with 42.9% presenting with severe disease. By the last follow-up, the proportion of patients with mild to moderate disease increased to 76.2%, while those with severe disease decreased to 23.8%. These results suggest an overall improvement in functional status in the cohort, reflecting the effectiveness of treatment strategies over time.

Given the overlap between NBD and other neurological conditions, like multiple sclerosis (MS), sarcoidosis, Sjögren’s syndrome, and primary CNS vasculitis, we took several steps to differentiate them in cases with ambiguous presentations, following established criteria [41]. For MS, we relied on several key differences: while both affect patients in the third or fourth decade with similar neurological symptoms, MS is more common in females and NBD in males. MS typically shows periventricular, posterior fossa, and corpus callosum lesions on MRI, whereas NBD presents large lesions in the brainstem, basal ganglia, and diencephalon. Additionally, CSF in MS often shows >95% oligoclonal bands (OCB), while NBD patients show <15% OCB.

This study is not without limitations. The retrospective design may introduce selection and reporting biases due to reliance on medical records. Underdiagnosis remains a concern, particularly for patients with mild neurological symptoms or overlapping presentations that could be misattributed to other conditions. Additionally, cultural and social factors in Lebanon may contribute to the under-reporting of symptoms such as genital ulcers, which are considered sensitive topics in the region. The study was also conducted during a period of significant socioeconomic challenges in Lebanon, including the recent economic crisis, which likely affected healthcare access, diagnostic follow-up, and treatment adherence. The affordability and availability of advanced diagnostic tools, such as MRI and cerebrospinal fluid analysis, may have been compromised, potentially impacting the accurate identification of NBD cases. Furthermore, our single-center study, while valuable for understanding local disease patterns, may not be generalizable to other regions. Finally, the small sample size of NBD patients limited our ability to perform statistical analyses or hypothesis testing.

Although this study was conducted in Lebanon, the insights gained are increasingly relevant on a global scale. With growing international migration and population mobility, Behçet’s Disease is being encountered more frequently in regions where it was once considered rare [42,43]. This highlights the importance of raising awareness among clinicians in non-endemic areas to improve early recognition and diagnosis, particularly of less common but serious manifestations like Neuro-Behçet’s Disease.

## 5. Conclusions

This study provides insights into the clinical and radiological characteristics, disease progression, and management of NBD withing a Lebanese cohort. Our findings underscore the high prevalence of non-parenchymal NBD and the more severe outcomes associated with parenchymal involvement. Notably, we observed gender dynamics that challenge the male predominance reported in other regions, with a balanced gender distribution and higher disease severity in female patients. The findings highlight the diagnostic challenges of NBD, the high prevalence of HLA-B51, and the impact of regional variations on clinical presentation. Limitations such as underdiagnosis and socioeconomic barriers highlight the need for improved diagnostic strategies and equitable healthcare access. Future multicenter studies are essential to enhance our understanding and management of this rare but debilitating condition.

## Figures and Tables

**Table 1:** Baseline characteristics, clinical manifestations, inflammatory blood markers, and HLA-B51 status of patients stratified by NBD status.

Variables	Neuro-Behçet’s Disease (*n* = 27)	Behçet’s Disease (*n* = 235)	Total (*n* = 262)
Baseline characteristics			
Age (years)	47.5 ± 13.5	43.6 ± 15.4	44.0 ± 15.3
Gender (female)	14 (51.9%)	112 (47.7%)	126 (48.1%)
Smoking history (current smoker)	14 (58.3%)	88 (47.6%)	102 (48.8%)
History of alcohol intake (*n* = 161)	4 (18.2%)	16 (11.5%)	20 (12.4%)
Educational level (*n* = 120) (college)	11 (78.6%)	73 (68.9%)	84 (70.0%)
Age of onset of NBD (years)	38 ± 13.6	NA	NA
Age of onset of BD (years)	32.3 ± 13.3	30.3 ± 14.2	30.5 ± 14.0
Time to NBD (years)	4.6 ± 7.7	NA	NA
Non-neurologic symptoms			
Oral ulcers	23 (85.2%)	191 (81.3%)	214 (81.7%)
Genital ulcers	19 (70.4%)	125 (53.2%)	144 (55.0%)
Uveitis	12 (44.4%)	100 (42.6%)	112 (42.7%)
GI involvement	4 (14.8%)	38 (16.2%)	42 (16.0%)
Arthritis or arthralgia	14 (51.9%)	102 (43.4%)	116 (44.3%)
Cutaneous involvement	11 (40.7%)	59 (25.1%)	70 (26.7%)
DVT	3 (9.2%)	27 (11.5%)	30 (11.5%)
Neurologic symptoms			
Headache	24 (88.9%)	34 (14.5%)	58 (22.1%)
Diplopia	3 (11.1%)	3 (1.3%)	6 (2.3%)
Dysphagia	1 (3.7%)	3 (1.3%)	4 (1.5%)
Dizziness	11 (40.7%)	9 (3.8%)	20 (7.6%)
Unsteadiness	9 (33.3%)	5 (2.1%)	14 (5.3%)
Seizure/epilepsy	5 (18.5%)	3 (1.3%)	8 (3.1%)
Weakness	17 (63%)	12 (5.1%)	29 (11.1%)
Paresthesia	6 (22.2%)	8 (3.4%)	14 (5.3%)
Cognitive/behavioral changes	7 (25.9%)	1 (0.4%)	8 (3.1%)
Psychosis	2 (7.4%)	2 (0.9%)	4 (1.5%)
Genetic and inflammatory blood markers			
HLA-B51 positive (*n* = 60)	4 (80.0%)	46 (83.6%)	50 (83.3%)
Elevated CRP pre-treatment (*n* = 42)	8 (61.5%)	17 (58.6%)	25 (59.5%)
Elevated ESR pre-treatment (*n* = 40)	8 (53.3%)	14 (56.0%)	22 (55.0%)
Elevated CRP post-treatment (*n* = 45)	3 (27.3%)	7 (20.6%)	10 (22.2%)
Elevated ESR post-treatment (*n* = 36)	3 (23.1%)	6 (26.1%)	9 (25%)

**Table 2:** Medical treatment received.

Variables	Neuro-Behçet’s Disease (*n* = 27)	Behçet’s Disease (*n* = 235)	Total (*n* = 262)
Corticosteroids	21 (77.8%)	114 (48.5%)	135 (51.5%)
Immunosuppressants			
Azathioprine	13 (48.1%)	40 (17%)	53 (20.2%)
Methotrexate	6 (22.2%)	17 (7.2%)	23 (8.8%)
Mycophenolate mofetil	5 (18.5%)	3 (1.3%)	8 (3.1%)
Cyclophosphamide	5 (18.5%)	1 (0.4%)	6 (2.3%)
Cyclosporine	2 (7.4%)	4 (1.7%)	6 (2.3%)
Biologic agents			
Infliximab	5 (18.5%)	26 (11.1%)	31 (11.8%)
Adalimumab	3 (11.1%)	34 (14.5%)	37 (14.1%)
Tocilizumab	1 (3.7%)	0	1 (0.4%)
Rituximab	2 (7.4%)	3 (1.3%)	5 (1.9%)
Immunomodulators			
Interferon alpha	0	3 (1.3%)	3 (1.1%)
Colchicine	16 (59.3%)	171 (72.8%)	187 (71.4%)
Apremilast	0	1 (0.4%)	1 (0.4%)
Antimalarials			
Chloroquine	0	2 (0.9%)	2 (0.8%)
Hydroxychloroquine	0	1 (0.4%)	1 (0.4%)

**Table 3:** Cutaneous manifestations and uveitis localization in NBD patients.

Variables	Count (%)
Skin findings	
Pseudo folliculitis	5 (45.5%)
Erythema nodosum	5 (45.5%)
Non specified erythematous skin rash	1 (9.1%)
Uveitis location	
Anterior	2 (25.0%)
Posterior	1 (12.5%)
Panuveitis	5 (62.5%)
Unilateral	5 (62.5%)
Bilateral	3 (37.5%)

**Table 4:** Baseline characteristics, clinical manifestations of NBD, and MRI findings of NBD patients stratified by mRS (mild to moderate vs. severe disability).

Variables	Mild to Moderate Disability (*n* = 20, 74.1%)	Severe Disability
		(*n* = 7, 25.9%)
Baseline characteristics		
Age (years)	46 ± 14.6	51.14 ± 9.8
Gender (female)	9 (45.0%)	5 (71.4%)
Smoking history (current smoker) (*n* = 24)	11 (61.1%)	3 (50.0%)
History of alcohol intake (*n* = 22)	4 (25.0%)	1 (16.7%)
Educational level (college) (*n* = 14)	9 (81.8%)	2 (66.7%)
Age of onset of BD (years)	31.3 ± 13.4	35.3 ± 13.7
Age of onset of NBD (years)	37.4 ± 14.5	39.8 ± 11.5
Behçet’s Disease duration (years)	7.5 ± 6.9	10.0 ± 11.5
Age of onset of NBD (years)	37.4 ± 14.5	39.8 ± 11.5
Time to NBD (years)	3.7 ± 7.3	7.6 ± 8.5
Non-neurologic symptoms		
Oral ulcers	17 (85.0%)	6 (85.7%)
Genital ulcers	15 (75.0%)	4 (57.1%)
Uveitis	7 (35.0%)	5 (71.4%)
GI involvement	6 (30.0%)	0
Arthritis or arthralgia	10 (50.0%)	4 (57.1%)
Cutaneous involvement	8 (40.0%)	4 (57.1%)
DVT	2 (10.0%)	1 (14.3%)
Neurologic symptoms		
Headache	19 (95.0%)	5 (71.4%)
Diplopia	2 (10.0%)	2 (28.6%)
Dysphagia	1 (5.0%)	0
Dizziness	8 (40.0%)	3 (42.9%)
Unsteadiness	6 (30.0%)	3 (42.9%)
Seizure/epilepsy	3 (15.0%)	2 (28.6%)
Weakness	13 (65.0%)	4 (57.1%)
Paresthesia	4 (20.0%)	2 (28.6%)
Cognitive/behavioral changes	4 (20.0%)	3 (42.9%)
Psychosis	2 (10.0%)	0
MRI findings		
Brainstem	6 (30.0%)	2 (28.6%)
Basal ganglia	2 (10.0%)	4 (57.1%)
Thalamus	3 (15.0%)	3 (42.9%)
Hypothalamus	1 (5.0%)	0
Internal capsule	3 (15.0%)	2 (28.6%)
Periventricular	6 (30.0%)	1 (14.3%)
Spinal cord	1 (5.0%)	1 (14.3%)
Sinus/vein thrombosis	2 (10.0%)	2 (28.6%)
Aneurysm	2 (10.0%)	0
Ischemia/infarcts	7 (35.0%)	2 (28.6%)
Cerebral bleeding	2 (10.0%)	0
Meningeal enhancement	3 (15.0%)	1 (14.3%)
Inflammatory blood markers		
Elevated CRP pre-treatment (*n* = 13)	4 (57.1%)	4 (66.7%)
Elevated ESR pre-treatment (*n* = 15)	4 (44.4%)	4 (66.7)
Elevated CRP post-treatment (*n* = 11)	1 (14.3%)	2 (50.0%)
Elevated ESR post-treatment (*n* = 13)	1 (11.1%)	2 (50.0%)

**Table 5:** Baseline characteristics, clinical manifestations, and MRI findings compared between parenchymal and non-parenchymal NBD.

Variables	Parenchymal (*n* = 8, 33.3%)	Non-Parenchymal (*n* = 16; 66.7%)
Baseline characteristics		
Age (years)	45.4 ± 15.2	47.3 ± 14.0
Gender (female)	3 (37.5%)	8 (50.0%)
Smoking history (current smoker) (*n* = 24)	1 (12.5%)	6 (46.2%)
History of alcohol intake (*n* = 22)	2 (33.3%)	3 (23.1%)
Educational level (college) (*n* = 14)	4 (100.0%)	6 (85.7%)
Age of onset of BD (years) (*n* = 21)	36.5 ± 12.3	28.3 ± 13.9
Age of onset of NBD (years) (*n* = 20)	39.0 ± 14.4	36.4 ± 14.1
Behçet’s Disease duration (years) (*n* = 15)	4.6 ± 8.2	11.0 ± 5.9
Time to NBD (years) (*n* = 20)	0.0 ± 5.8	6.4 ± 7.9
Non-neurologic symptoms		
Oral ulcers	7 (87.5%)	13 (81.3%)
Genital ulcers	5 (62.5%)	12 (75.0%)
Uveitis	5 (62.5%)	6 (37.5%)
GI involvement	1 (12.5%)	5 (31.3%)
Arthritis or arthralgia	2 (25.0%)	10 (62.5%)
Cutaneous involvement	4 (50%)	7 (43.8%)
DVT	0	2 (12.5%)
Neurologic symptoms		
Headache	6 (75.0%)	15 (93.8%)
Diplopia	2 (25.0%)	2 (12.5%)
Dysphagia	0	1 (6.3%)
Dizziness	2 (25.0%)	9 (56.3%)
Unsteadiness	3 (37.5%)	6 (37.5%)
Seizure/epilepsy	2 (25.0%)	3 (18.8%)
Weakness	6 (75.0%)	10 (62.5%)
Paresthesia	1 (12.5%)	5 (31.3%)
Cognitive/behavioral changes	3 (37.5%)	4 (25.0%)
Psychosis	0	2 (12.5%)
mRS score		
Mild to moderate disability	5 (62.5%)	13 (81.3%)
Severe disability	3 (37.5%)	3 (18.8%)

**Table 6:** MRI findings in patients with parenchymal vs. non-parenchymal NBD.

MRI Findings	Count (%)
Non-parenchymal NBD (*n* = 16)	
Sinus/vein thrombosis	5 (31.3%)
Ischemia/infarcts	9 (56.3%)
Aneurysm	2 (12.5%)
Meningeal enhancement	4 (25%)
Cerebral bleeding	2 (12.5%)
Parenchymal NBD (*n* = 8)	
Brainstem	6 (75.0%)
Basal ganglia	3 (37.5%)
Thalamus	5 (62.5%)
Hypothalamus	1 (12.5%)
Internal capsule	3 (37.5%)
Periventricular	2 (25.0%)
Spinal cord	1 (12.5%)

**Table 7:** Disease progression in NBD patients, stratified by mRS score.

mRS Score (0–6)	At Presentation (*n* = 21)	At Last Follow-Up (*n* = 21)
0	0	4 (19%)
1	5 (23.8%)	5 (23.8%)
2	2 (9.5%)	4 (19%)
3	5 (23.8%)	3 (14.3%)
4	7 (33.3%)	2 (9.5%)
5	1 (4.8%)	1 (4.8%)
6	1 (4.8%)	2 (9.5%)
Mild to moderate (0–3)	12 (57.1%)	16 (76.2%)
Severe (4–6)	9 (42.9%)	5 (23.8%)

## Data Availability

Data are available upon reasonable request.

## Supplementary Materials

The following supporting information can be downloaded at https://www.mdpi.com/article/10.3390/jcm14082543/s1: Table S1. Modified Rankin Scale (mRS) score description.

## Abbreviations

The following abbreviations are used in this manuscript:

BD	Behçet’s Disease
HLA	Human Leukocyte Antigen
ICBD	International Criteria for Behçet’s Disease
ISG	International Study Group
NBD	Neuro-Behçet’s Disease
CNS	central nervous system
BD-NI	BD without neurological involvement
mRS	modified Rankin Scale
DVT	deep-vein thrombosis
CVT	cerebral venous thrombosis
