# Supplementary material for: Clinical Characteristics, MRI Findings, Disease Progression, and Management of Neuro-Behçet’s Disease: A Retrospective Study in Lebanon
Source: J Clin Med. 2025 Apr 8;14(8):2543. doi: 10.3390/jcm14082543 (PMC12027848; doi:10.3390/jcm14082543)
Supplement: Supplementary file 1 [file jcm-14-02543-s001.zip › jcm-3507659-supplementary.pdf]

*Table S1: Modified Rankin Scale (mRS) Score Description*

| MRS | Description |
|---|---|
| 0 | No symptoms |
| 1 | No significant disability, despite symptoms; able to perform all usual duties and activities |
| 2 | Slight disability: unable to perform all previous activities but able to look after own affairs without assistance. |
| 3 | Moderate disability: requires some help, but able to walk without assistance. |
| 4 | Moderately severe disability; unable to walk nor attend to own bodily needs without assistance. |
| 5 | Severe disability; bedridden, incontinent and requires nursing care and attention |
| 6 | Dead |
